# Acquiring and Producing Sentences: Whether Learners Use Verb-Specific or Verb-General Information Depends on Cue Validity

**DOI:** 10.3389/fpsyg.2016.00404

**Published:** 2016-03-23

**Authors:** Malathi Thothathiri, Michelle G. Rattinger

**Affiliations:** Department of Speech and Hearing Science, The George Washington UniversityWashington, DC, USA

**Keywords:** sentence production, statistical learning, verb bias, cue validity, artificial language

## Abstract

Learning to produce sentences involves learning patterns that enable the generation of new utterances. Language contains both verb-specific and verb-general regularities that are relevant to this capacity. Previous research has focused on whether one source is more important than the other. We tested whether the production system can flexibly learn to use either source, depending on the predictive validity of different cues in the input. Participants learned new sentence structures in a miniature language paradigm. In three experiments, we manipulated whether individual verbs or verb-general mappings better predicted the structures heard during learning. Evaluation of participants’ subsequent production revealed that they could use either the structural preferences of individual verbs or abstract meaning-to-form mappings to construct new sentences. Further, this choice varied according to cue validity. These results demonstrate flexibility within the production architecture and the importance of considering how language was learned when discussing how language is used.

## Introduction

As speakers, we do not simply repeat the same set of sentences every day but produce new sentences to describe events in the world as well as our internal thoughts. Thus, an important aspect of learning to talk is knowing how to generate novel utterances that obey the grammatical rules of language. A persistent question for research is whether the sentence production system accomplishes this feat by preferentially encoding and using knowledge about how specific verbs are used in different types of sentences or knowledge about how broad meanings are mapped to different word orders independent of specific verbs. Many theories of language acquisition and language production give priority to one of the two sources of structural information. Here we use a cue validity framework to test the idea that the mental architecture for learning to produce sentences might flexibly adapt to use either type of information depending on statistical regularities in the input.

Sentence production involves converting a thought into a sequence of sounds. There is consensus that this process includes the steps of conceptualization, grammatical encoding, and phonological encoding, before articulation (Bock and Levelt, 1994). The focus of this paper is grammatical encoding (Levelt, 1989). This is the stage at which different words are assigned to different grammatical functions (e.g., subject, direct object) and assembled into an ordered sequence. The challenge for the speaker is that any given language contains multiple possible word orders. For example, when describing the scenario of a dog running after a cat, an English speaker could say that “the dog chased the cat” or that “the cat was chased by the dog.” Similarly, when talking about Lily transferring a container of flowers to John, a speaker could say either that “Lily gave John the vase” or that “Lily gave the vase to John.” In contrast, “The dog chased the cat a ball” and “Lily gave John” are ungrammatical. Speakers seldom, if at all, make such errors, suggesting that they implicitly know that a given word order can be used in some scenarios but not others. What are the constraints that enable speakers to produce novel grammatical utterances while avoiding ungrammatical utterances?

One possible constraint on word order, illustrated in part by the above examples, is the main verb in the sentence. “Chase” describes a transitive action and requires two nouns in the sentence (the entity doing the chasing and the entity being chased) while “give” describes a transfer action and requires three nouns in the sentence (the entity giving, the thing that is given, and the entity receiving). Additionally, some verbs appear to be idiosyncratic with respect to the types of sentences in which they appear. For example, native English listeners judge “Bob donated the art collection to the museum” as being more acceptable than “Bob donated the museum the art collection.” Such observations have led to theories that assign importance to individual verbs in how syntactic knowledge is learned, represented and used, within linguistics (Chomsky, 1981, 1986; Kaplan and Bresnan, 1982; Pollard and Sag, 1994; Ferreira, 2000), language acquisition (Tomasello, 2000, 2003), language production (Bock and Levelt, 1994), and language comprehension (MacDonald et al., 1994; Trueswell and Tanenhaus, 1994). For example, it has been proposed that children initially learn to speak by using verb-specific sentence frames and that generalizations that apply to different verbs only emerge later (Tomasello, 2000, 2003). During production, it is postulated that speakers first retrieve the lemma associated with the verb, which provides the syntactic information necessary to construct a sentence (Bock and Levelt, 1994). Similarly, dominant constraint-based theories of language comprehension assume that listeners use the syntactic information associated with individual lexical items for sentence interpretation (MacDonald et al., 1994; Trueswell and Tanenhaus, 1994). We refer to such theories collectively hereafter as “lexicalist.” Although the different theories vary widely in many respects (e.g., how much syntactic knowledge is assumed to be innate vs. learned), they share the idea that individual lexical items, particularly verbs, play an important role in sentence production and comprehension.

A contrasting viewpoint is provided by theories that emphasize the role of abstract or verb-independent structural knowledge. Such abstract knowledge is postulated in part based on observations that people can produce and understand novel verbs (e.g., the first use of “google” as a verb as in “She googled the directions to his place”), use known verbs in unusual sentence structures (e.g., “She smiled herself an upgrade” Goldberg, 2003), and show structural or syntactic priming between sentences containing different verbs (e.g., see Thothathiri and Snedeker, 2008a,b). These capacities necessarily require that the language user represent and use mappings between meaning and sentence form that are not tied to individual lexical items. As with lexicalist theories above, “abstractionist” theories have been proposed within language acquisition (Fisher, 2002a,b), language production (Bock, 1990; Bock and Loebell, 1990; Konopka and Bock, 2009), and language comprehension (Bencini and Goldberg, 2000; Kaschak and Glenberg, 2000). These theories share the idea that abstract structural knowledge, which is not tied to specific lexical items, is used routinely to understand and produce sentences.

The balance between lexical conservatism and broad generalization has been investigated extensively within language acquisition. Different theories have attempted to account for the balance in different ways. For example, Pinker (1989) proposed that children initially rely on innate “broad-range” linking rules that tie abstract, non-verb-specific meanings to sentence structures. This enables correct generalization of structures from one verb to another but also leads to overgeneralization errors. Under the model proposed by Pinker (1989), children can prune such errors later during development by acquiring “narrow range” linking rules that tie specific classes of verbs to specific structures based on meaning. For example, children could learn that while many dative verbs alternate between the double-object and prepositional-object constructions (e.g., “Lily gave John the vase,” “Lily gave the vase to John”), verbs that encode the meaning of “continuous imparting of force” (e.g., carry) are not (entirely) grammatical in the double-object construction. This approach is called “semantic bootstrapping” because knowledge about the meanings of verbs is used to restrict structural choices. It has come under criticism because crosslinguistically, the structures that verbs appear in are not entirely predictable from verb semantics (see e.g., Bowerman and Brown, 2008). An alternative distributional or statistical learning approach proposes that speakers learn to produce utterances by tracking the statistical distribution of different lexical items in different constructions in the input (see e.g., Twomey et al., 2014). This approach has gained momentum in the last two to three decades due to demonstrations of impressive statistical learning abilities in infants, children, and adults. In particular, studies under this approach have investigated how learners deal with structural variation associated with different syntactic categories (Nouns: Hudson Kam and Newport, 2005, 2009; Smith and Wonnacott, 2010; Wonnacott, 2011. Verbs: Wonnacott et al., 2008, 2012; Perek and Goldberg, 2015). Here we focus on the learning of verb-related distributional information.

Broadly speaking, empirical evidence from statistical learning experiments suggests that learners can acquire and use both verb-specific and verb-independent information extracted from the input. In one study, adult participants learned a miniature language containing novel vocabulary and two novel syntactic structures (Wonnacott et al., 2008). During training, the researchers manipulated each individual verb’s relative preference for one structure vs. the other. Subsequent production and comprehension tasks showed that participants tended to use verbs in the structures with which they were statistically associated during training, consistent with the acquisition of verb-specific syntactic knowledge (Wonnacott et al., 2008). These findings agree with results from a number of paradigms, which show that individual verbs’ statistical associations with different sentence structures (hereafter “verb bias”) influence language comprehension and production in both adults and children (e.g., Garnsey et al., 1997; Stallings et al., 1998; Snedeker and Trueswell, 2004; Wilson and Garnsey, 2009). Empirical support also exists for the alternate perspective. Wonnacott et al. (2012) taught 5-year-old children a novel sentence form that corresponded to a novel meaning. Subsequent to training, children were tested on comprehension and production of the novel construction. Critically, a subset of the test trials involved new verbs not heard during training. Children successfully generalized from verbs heard during training to verbs heard during test, demonstrating the learning and use of verb-independent form-meaning mappings (Wonnacott et al., 2012, See also Casenhiser and Goldberg, 2005). Results from other paradigms similarly indicate that adults and children use abstract structural knowledge during language comprehension and production (e.g., Gertner et al., 2006; Thothathiri and Snedeker, 2008a,b; Konopka and Bock, 2009; Rowland et al., 2012).

Although debates within language acquisition and language processing are often framed in exclusionary terms, the two types of structural knowledge are not mutually exclusive (Thothathiri and Snedeker, 2008a). This is explicitly acknowledged by linguistic frameworks that have suggested a continuum of abstraction rather than a dichotomy (e.g., Jackendoff, 2002; Goldberg, 2003). A recent computational demonstration comes from a connectionist model of learning to talk that incorporated multiple cues, including lexically specific knowledge about word order and abstract mappings between event roles and sentential positions, which operated together during language acquisition (Chang et al., 2006). The model successfully mimicked aspects of linguistic behavior that have been observed in adults and children. This is proof of principle that the two sources of structural information can co-exist in learners’ minds. The important remaining question is how the language system weights the two types of information in different situations (Thothathiri and Snedeker, 2008a; Konopka and Bock, 2009; Rowland et al., 2012). While previous studies have documented the use of one or the other type of information, principled investigations of what guides the speaker to use one source vs. the other are rare [but see Perfors et al. (2010) for a hierarchical Bayesian account]. One exception is a recent study by Perek and Goldberg (2015). In this study, participants learned a language containing two novel word orders (Subject-Object-Verb and Pronoun-Subject-Verb). As in the Wonnacott et al. (2008) study, the researchers manipulated the statistical preference of different verbs for one word order or the other during training and showed that participants were able to learn and use such verb-specific information. However, participants’ production during test also depended on the discourse context. In the full-noun-phrase-biased context, participants were asked to describe scenes in response to the prompt “what happened here?” In the pronoun-biased context, the prompt was “what happened to the <animal>?” The key finding was that participants tended to use the construction that was discourse appropriate (e.g., Pronoun-Subject-Verb in the pronoun-biased context) even if that involved overriding verb-specific statistical preferences. Thus, Perek and Goldberg (2015) showed that speakers’ reliance on verb-specific or verb-general information could be shifted by the context under which language production occurs. In the present study, we tackle the problem exclusively from the perspective of how language input affects language production. Without manipulating discourse context or nudging the speakers in any way during the test phase, we ask whether different statistical properties encountered during the training phase alone can predictably lead speakers to rely on one source of information or the other.

A critical insight from connectionist frameworks is that the architecture of language processing is influenced by how language is learned (Plaut et al., 1996; Joanisse and Seidenberg, 1999; Gordon and Dell, 2003; Chang et al., 2006). The neural networks in these models do not just learn specific instantiations within a fixed architecture but reorganize and create interesting divisions of labor between different processes that capture behavioral dissociations observed in humans (Plaut et al., 1996; Chang, 2002; Gordon and Dell, 2003). These divisions of labor arise from competition between cues during learning such that reliable cues come to influence processing more than less reliable cues. We adopted a similar perspective and asked whether the human sentence production system can flexibly prioritize different sources of structural information under different learning conditions. We used a cue validity framework (Bates and MacWhinney, 1987; Goldberg et al., 2005; MacWhinney, 2013) to set up and test hypotheses about learning to produce sentences. In three experiments, we trained participants in a miniature language containing higher cue validity for verb-specific over abstract structural information, or a miniature language containing the opposite pattern. After training, we examined participants’ sentence production in the new language to evaluate whether they relied more on verb-specific or verb-independent knowledge. If the language learning architecture is flexible, we would expect different degrees of reliance on the two sources in different types of languages. Alternatively, if the sentence production system has a fixed architecture that prioritizes one source of information over the other, we would expect no effect of the type of language. The results have implications for theories of language acquisition and processing.

## Experiment 1

In Experiment 1, participants were trained in a miniature language that contained two novel structures for describing transitive events (**Table 1**). The two structures both began with the verb. They differed in whether the agent of the transitive action was placed before the patient (hereafter agent-before-patient or AP order) or the patient before the agent (hereafter patient-before-agent or PA order). During training, we manipulated verb bias such that some verbs appeared solely in AP order (hereafter AP-only verbs), some solely in PA order (hereafter PA-only verbs), and others equally in the two orders (hereafter Alternating or Alt verbs). After training, participants were asked to describe new events. We analyzed the proportions of AP and PA orders produced in different conditions.

**Table 1:** Miniature language input provided to participants in the three experiments.

Experiment	Event type(s)	Sentence structures	Bias manipulations
Experiment 1	Transitive Transitive	AP order: Verb-Agent-Patient PA order: Verb-Patient-Agent-ka	12 verbs: 4 AP-only, 4 PA-only, 2 Alternating, 2 Synonymous
Experiments 2 and 3	Instrument Modifier	AP order: Verb-Agent-Patient-Object PA order: Verb-Patient-Agent-Object-ka	12 verbs: 4 AP-only, 4 PA-only, 2 Alternating, 2 Synonymous

If learners exclusively encode and use abstract structural representations, we would expect no differences between the different bias conditions. This is because all verbs in this experiment referred to transitive actions and this meaning could be mapped to either the AP or the PA order. In contrast, if learners encode and use verb-specific structural associations, we would expect systematic differences between bias conditions, with the lowest PA order proportion for AP-only verbs (0% PA order during training) and the highest proportion for PA-only verbs (100% PA order during training), and an in-between proportion for Alt verbs (50% PA order during training). We sought to replicate the results from a closely related previous study, which showed an effect of verb bias on participants’ language production (Wonnacott et al., 2008). Further, we attempted to address a potential confound in that study and explore the nature of the statistical associations acquired by the participants. In the previous study, the verb bias effects could have arisen from participants learning the associations between sentence structures and individual verb lemmas (e.g., the novel verb “fenk”) or the associations between sentences structures and the depicted action meanings (e.g., hitting). To tease apart the two possibilities, we included synonymous verbs in the current study. The synonymous verbs described the same physical action but were associated with different lemmas and word orders (cf. *I like that* and *That pleases me* in English). This meant that the action was linked 50–50 to AP and PA order but the individual verbs were associated 100% with a single sentence structure. Therefore, if participants learned action-structure associations only, we would expect the synonymous verbs to behave like Alt verbs. Alternatively, if participants learned lemma-structure associations, we would expect the synonymous verbs to pattern according to their statistical biases.

To summarize, the primary goals of Experiment 1 were to replicate previous findings of verb bias effects and to explore the nature of the underlying representations that lead to such effects. In the context of learning sentences, cue validity is defined as the conditional probability of a sentence structure given the cue. The present study manipulated two types of cues, namely individual verbs and abstract mappings between meaning and structure. In Experiment 1, transitive events were described equally often using the AP or the PA structure, leading to 50% cue validity for abstract meaning-to-structure mappings. In comparison, the average cue validity for individual verbs was higher (100% for AP-only and PA-only verbs, and 50% for Alt verbs). Therefore, a cue validity framework would predict that learners would encode and use individual verb biases for production under these conditions (consistent with the previous results and our predictions for this experiment).

### Materials and Methods

#### Participants

Thirty right-handed native English speakers from the Washington, D.C. area completed all portions of the study (18–24 years, mean = 19.86, 24 female). Eleven were excluded because they did not produce any verb-first structures, leaving a total of nineteen in the final analyses. All participants gave consent under a protocol approved by the Institutional Review Board at The George Washington University.

#### Stimuli

The miniature language was adapted from Wonnacott et al. (2008). The language contained English nouns, but novel verbs and sentence structures. The 12 novel verbs (*fenk, flern, frag, glim, gofe, gund, parn, pelk, pruf, semz, sig, stoom*) were used to describe transitive actions. Ten of the verbs described 10 distinct actions (hit, hug, jump on, kiss, lift, poke, pull, push, shake, tap). Two other synonymous verbs described the same physical action (stroke). The verb forms corresponding to the different meanings were counterbalanced across five lists. Each participant was pseudorandomly assigned to a list.

The language contained two novel structures [verb agent patient, verb patient agent particle (“ka”) Wonnacott et al. (2008)]. The two structures both began with the verb. Both encoded a transitive meaning. They differed in whether they contained AP or PA order. During training, we manipulated verb bias. Four of the 12 verbs appeared only in AP order (AP-only verbs), four only in PA order (PA-only verbs), and two equally in both orders (Alt verbs). The remaining two verbs were the synonymous verbs – one appeared only in AP order, the other only in PA order.

**FIGURE 1:**
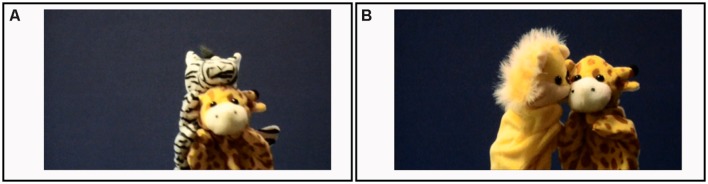
**Example stills from training videos in Experiment 1. (A)** Action = zebra jumps on giraffe, accompanied by a spoken AP order sentence (e.g., *pelk zebra giraffe*). **(B)** Action = lion kisses giraffe, accompanied by a spoken PA order sentence (e.g., *fenk giraffe lion ka*).

Actions were depicted using videos involving puppets. Each video was roughly 4 s long and showed one puppet acting on another. During training, each video was accompanied by a sentence in the miniature language (**Figure 1**). A female native English speaker recorded each word at different sentential positions. These recordings were combined to form different sentences using the SoX command line utility (sox.sourceforge.net).

#### Procedure

Stimuli were presented using E-prime. Each participant underwent three training sessions and one testing session. The three training sessions were identical. They were conducted on three separate days, with no more than 3 days between sessions. In each training session, participants watched 144 videos where animal puppets acted on one another (e.g., zebra jumping on giraffe). Each video was accompanied by a pre-recorded sentence (e.g., *pelk zebra giraffe*). Participants were instructed to listen to the sentence, repeat it out loud, and then press any key to move on to the next trial. The 144 trials consisted of 48 trials with AP-only verbs, 48 trials with PA-only verbs, 24 trials with Alt verbs, and 24 trials with the synonymous verbs. Each Alt and synonymous verb appeared 12 times per session. Half of the AP-only and PA-only verbs appeared at a lower frequency (6 times per session) and half at a higher frequency (18 times per session). This variation in verb frequency [similar to the manipulation in Wonnacott et al. (2008)] was included to investigate the potential effects of frequency on learning and using verb bias. Previous results had suggested that participants might be more likely to adhere to verb bias with high frequency verbs [see Wonnacott et al. (2008) for discussion]. However, in the current study, we found no main effects of or interactions with frequency. Therefore, all results are reported collapsed across this variable.

After training, participants returned within 3 days for a final testing session. During the test, they watched new videos and described them using the miniature language. Critically, the test videos involved new animal puppets (and correspondingly new nouns) compared to training (**Appendix Table A1**). Thus, participants could not rely on memorized word combinations from training. They had to generate new sentences, which allowed us to evaluate their syntactic knowledge.

The test began with a familiarization phase where participants were asked to name the new animal puppets one by one. The experimenter provided feedback if necessary. Subsequently on each test trial, they saw a miniature language verb to use in describing the upcoming video, watched the video clip (4000 ms), and then provided a verbal response (maximum 5000 ms). Forty-eight test trials were split across two blocks (16 AP-only, 16 PA-only, 8 Alt, 8 synonymous). The test session lasted approximately 20–30 min.

#### Coding

We transcribed participants’ responses to the test videos and coded the structures as containing AP or PA order. Only trials containing the correct verb and the correct two nouns were classified into the AP/PA categories. All other responses, including incorrect verb or noun(s), repeats, and incomplete responses, were classified as “Other.” For four randomly selected participants, a different coder transcribed and coded the responses. There was high inter-rater reliability (Cohen’s Kappa = 0.96).

#### Analyses

Because the dependent variable (structure produced = AP or PA order) was binomial, we used mixed effects logistic regression (glmer function with the bobyqa optimizer. lme4 version 1.1.10 in R. Bates et al., 2015). Two separate models addressed the two questions posed in this experiment. The first model examined whether verb bias affected the proportions of AP/PA orders produced with AP-only, Alt and PA-only verbs. If the proportions vary according to statistical bias, we would expect a linear trend in PA order production (AP-only < Alt < PA-only). The model contained the fixed Bias factor and the maximal random effects structure warranted by the design, including random intercept and slope by participant and random intercept by item (Barr et al., 2013). The bias factor was coded using orthogonal polynomial coding. The second model examined whether Syn-AP verbs (synonymous verbs that only appeared in AP order during training) patterned similarly to AP-only verbs or to Alt verbs. The bias factor was dummy-coded with the Syn-AP condition as the reference level.

### Results

**FIGURE 2:**
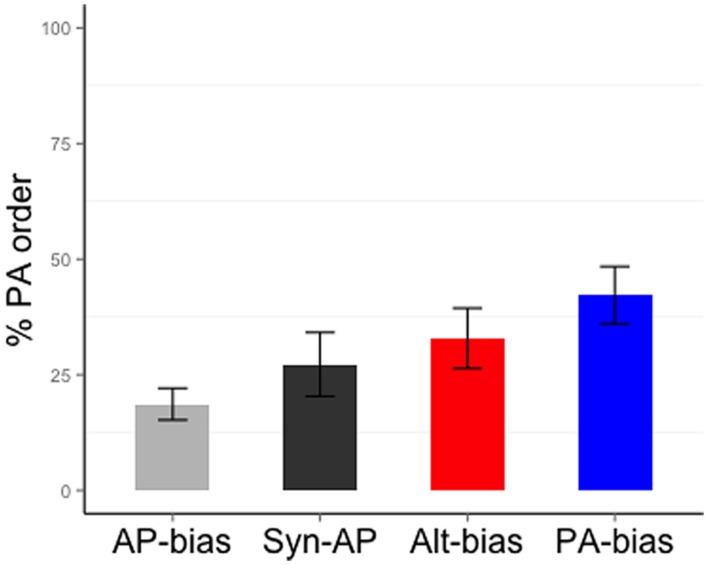
**Proportion of PA order responses produced with different verb types in Experiment 1**.

Participants showed an overall preference for AP order (65.24% of the responses contained AP order; 28.18% contained PA order, 6.58% were other, *mu* = 50, *t*(18) = 3.62, *p* = 0.002). The proportion of PA order [=PA responses/(PA responses + AP responses)^∗^100] for each verb bias condition is shown in **Figure 2** (PA order proportion for AP-only = 18.66, Syn-AP = 27.26, Alt = 32.89, PA-only = 42.22). Note that these proportions are reported solely for ease of interpretation; all statistical analyses were performed in log-odds space. **Table 2** shows the results from the mixed model analyses. In the model containing AP-only, Alt and PA-only verbs, there was a significant linear trend that was consistent with verb bias (AP-only < Alt < PA-only. Wald’s *Z* = 2.51, *p* = 0.01). In the model comparing Syn-AP to AP-only and Alt verbs, neither contrast was significant (Syn-AP vs. AP-only: Wald’s *Z* = 0.05, *p* = 0.96; Syn-AP vs. Alt: Wald’s *Z* = 1.04, *p* = 0.30). Note: because a substantial number of participants were excluded in this experiment, we conducted the same analyses as above with all subjects included. As in the original analysis, there was a significant AP-only < Alt < PA-only linear trend, consistent with verb bias (Wald’s *Z* = 2.08, *p* = 0.04). Syn-AP verbs did not differ from AP-only verbs (Wald’s *Z* = 0.68, *p* = 0.49) but they were marginally different from Alt verbs (Wald’s *Z* = 1.96, *p* = 0.05). Thus, we confirmed the AP-only < Alt < PA-only pattern, and the non-significant difference between Syn-AP and AP-only verbs, in the entire sample.

**Table 2:** Mixed models of the likelihood of a PA order response in Experiment 1.

	Estimate	*SE*	Wald *Z*	*P*
**AP-only < Alt < PA-only**				
*Fixed effects*				
Intercept	-1.29	0.40	-3.19	0.001
Linear	0.90	0.36	2.51	0.01
Quadratic	0.04	0.47	0.09	0.93
**Syn-AP vs. AP-only and Alt**				
Fixed effects				
Intercept	-1.85	0.71	-2.62	0.009
AP-only	0.03	0.63	0.05	0.96
Alt	0.74	0.72	1.04	0.30

### Discussion

Experiment 1 replicated essential aspects of previous findings on verb bias effects during sentence production (Wonnacott et al., 2008). Participants were trained in a miniature language containing two alternative word orders for describing transitive events. We manipulated the statistical bias of different verbs for different structures. Subsequently, participants’ descriptions of new events, which could not rely on memorization, showed that the likelihood of PA order varied according to verb bias. Specifically, the likelihood of PA order showed a linear AP-only < Alt < PA-only trend. We augmented the design of the previous study by testing sentence production with a synonymous verb, whose lemma was linked exclusively to AP order but whose associated action was linked equally to AP and PA orders. Likelihood of PA production for this verb was indistinguishable from AP-only verbs both in the analysis of subjects who passed the inclusion criteria and in the analysis of all subjects. The comparison between the synonymous verb and Alt verbs was not significant in the former analysis but was marginally significant in the latter. This tentatively suggests that production with the Syn-AP verb was more similar to AP-only than Alt verbs and that learners encoded the structural preferences of lemmas and not just action-structure linkages.

The findings corroborate a plethora of previous evidence for lexically specific constraints on language processing (e.g., Garnsey et al., 1997; Snedeker and Trueswell, 2004). They fit quite naturally within constraint-based lexicalist frameworks, which assume rich lexical representations that contain information about how any given word can combine with other words in a sentence (MacDonald, 1994; MacDonald et al., 1994; Trueswell and Tanenhaus, 1994; Trueswell, 1996). Under these frameworks, language users represent and use linkages between individual verbs and structural configurations (Trueswell and Kim, 1998; See also Pickering and Branigan, 1998) for a similar proposal within a structural priming context). The strengths of the links depend on frequency of co-occurrence. Thus, verbs experienced often in one structural configuration (e.g., PA-only verbs in PA order) would likely be used in that configuration later on. Similarly, Chang et al. (2006) connectionist model learns to talk in part by learning how to sequence individual verbs in sentences. This makes the model sensitive to lexically specific structural preferences, particularly early on during language acquisition (Chang et al., 2006). In sum, verb bias effects such as those found here are consistent with dominant models of language learning and processing, which assume that the human language system^1^ is capable of encoding and using rich information about how specific words are used.

In the language used in Experiment 1, verbs were, on average, highly predictive of the sentence structures heard during training. Ten out of the twelve verbs (all except Alt) predicted the correct sentence structure 100% of the time. In contrast, event structure correctly predicted which structure would be heard only 50% of the time. In this context of higher predictive validity of verb cues than abstract meaning cues, we expected and found effects of verb bias. These effects are consistent with but do not exclusively support the cue validity framework. This is because the results are also consistent with a production architecture that prioritizes verb-specific information. Subsequent experiments therefore tested whether learners come to rely on an alternate cue namely, meaning-to-structure mappings, if statistical regularities in the input favor that cue. These experiments used a language in which two different structures each referred to two different types of events. Unlike Experiment 1, event meaning was 100% predictive of sentence structure. The verb bias manipulations stayed the same. In this altered learning context, would participants show greater sensitivity to meaning-to-form mappings than to verb-specific structural preferences?

## Experiment 2

The miniature language in this experiment contained two word orders that were associated with two different types of events (instrument and modifier). Instrument events involved the agent acting on the patient using an instrument (e.g., monkey pinching giraffe using a clip). During training, these were associated exclusively with AP order (e.g., *gund monkey giraffe clip*). Modifier events involved the agent acting on the patient who was holding the object (e.g., monkey brushing donkey who is holding paper). During training, these were associated exclusively with PA order (e.g., *flern donkey monkey paper ka*). Thus, type of event was 100% predictive of the sentence structures heard during training. The verb bias manipulations were the same as before, with some verbs appearing exclusively in AP order, some exclusively in PA order, and others equally in both (**Table 1**).

As in Experiment 1, participants were asked to describe new videos during the test phase. Verbs were only tested with the event type(s) with which they were associated during training (i.e., event type and verb bias were *not* fully crossed). This meant that AP-only, Syn-AP, and PA-only verbs were only tested with one type of event, and Alt verbs were tested with both event types. Therefore, Alt verbs provide the strongest test of whether participants relied on meaning-to-form mappings instead of verb-specific structural preferences. If learners successfully encode and use meaning-to-form mappings, we would expect proportion of PA order to be significantly different when Alt verbs are used to describe modifier events and when the same verbs are used to described instrument events. Alternatively, if learners fail to employ meaning-to-form mappings and rely instead on verb-specific structural preferences, we would expect no difference between the two event types.

Examination of a linear verb-bias trend akin to Experiment 1 was not possible in Experiment 2 because AP-only and PA-only verbs were tested with distinct event types, which resulted in conflation between event type and verb bias. However, we were able to examine whether production differed according to verb bias *within each event type.* For modifier events, we compared PA-only and Alt verbs (these were the only two verb types that appeared with these events). For instrument events, we compared AP-only, Alt and Syn-AP verbs (again, these were all the verb types that appeared with these events). If verb bias has an effect on sentence production despite the fact that this language contained a more valid cue to structure, we would expect systematic variation between the different conditions within each event type. Alternatively, if participants preferentially rely on meaning-to-form mappings instead of verb-specific information, we would expect no such differences.

Note that as in Experiment 1, the two word orders in Experiment 2 differed in the relative positions of the agent and the patient. We kept the position of the object the same in both structures. Although this created a somewhat unusual word order for the structure used to describe modifier events wherein the modifier was separated from the modified noun (cf. *The man answered the door naked*), it allowed us to ensure that the word order differences present in the input were comparable to the differences in Experiment 1. Additionally, it could be argued that the presence of a modifier, in whichever position, is infelicitous in contexts containing a single possible referent. However, to preview our results, we show that participants successfully learned to produce this modifier. This supports our claim that the language production system can flexibly learn patterns that are present in the input.

### Materials and Methods

#### Participants

Eighteen right-handed native English speakers from the Washington, D.C. area completed all portions of the study (18–24 years, mean = 19.61, 11 female). One participant did not produce verb-first sentence structures and was excluded, leaving a total of seventeen in the final analyses.^2^ All participants gave consent under a protocol approved by the Institutional Review Board at The George Washington University. None had participated in Experiment 1.

#### Stimuli

**FIGURE 3:**
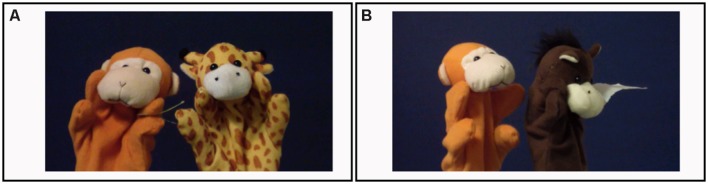
**Example stills from training videos in Experiment 2. (A)** Action = monkey pinches giraffe using a clip, accompanied by a spoken AP order sentence (e.g., *gund monkey giraffe clip*). **(B)** Action = monkey scratches donkey holding paper on the back, accompanied by a spoken PA order sentence (e.g., *flern donkey monkey paper ka*).

The verb forms, agents and patients were the same as in Experiment 1. Each visual scene and training sentence now additionally included an object. There were a total of eleven objects (ball, clip, cup, fan, feather, flower, paper, pencil, sponge, straw, tweezers). See **Figure 3** for examples. The depicted actions were brush, hit, knock down, pinch, point, poke, pull, scratch, stroke, tap and tickle. The verb forms corresponding to different meanings were counterbalanced across five lists. Each participant was pseudorandomly assigned to a list.

As before, four of the twelve verbs appeared only in AP order (AP-only), four only in PA order (PA-only), and two equally in the two orders (Alt). Also as before, there were two synonymous verbs, each associated with a different event type and order.

#### Procedure

Training and test procedures were identical to Experiment 1 except for one minor modification during test: after completing their verbal response for a trial, participants were permitted to advance to the next trial by hitting the spacebar, rather than wait for the remainder of the maximum trial duration. On each of 48 test trials (16 AP-only, 16 PA-only, 8 Alt, and 8 Syn-AP), participants saw a verb to use in describing the upcoming video, watched the video clip, and then provided a verbal response. As explained before, verbs were only tested with the event type(s) with which they were associated during training.

#### Coding

As in Experiment 1, we transcribed and coded participants’ responses as AP order, PA order, or Other. Responses had to contain the correct verb, agent and patient and an additional object in order to be counted as AP or PA. For four randomly selected participants, a different coder transcribed and coded the responses. There was high inter-rater reliability (Cohen’s Kappa = 0.94).

#### Analyses

For Alt verbs, we explored whether there was an effect of event type. The model contained the fixed effect of Event Type along with random effects. The event type factor was dummy-coded with Instrument events as the reference level. For the analysis of verb bias within modifier events, the model contained the fixed effect of verb bias along with random effects. The bias factor was dummy-coded with Alt verbs as the reference level. For instrument events (AP-only, Syn-AP, and Alt verbs), the model was similar to the one above, with AP-only verbs as the reference level.

### Results

**FIGURE 4:**
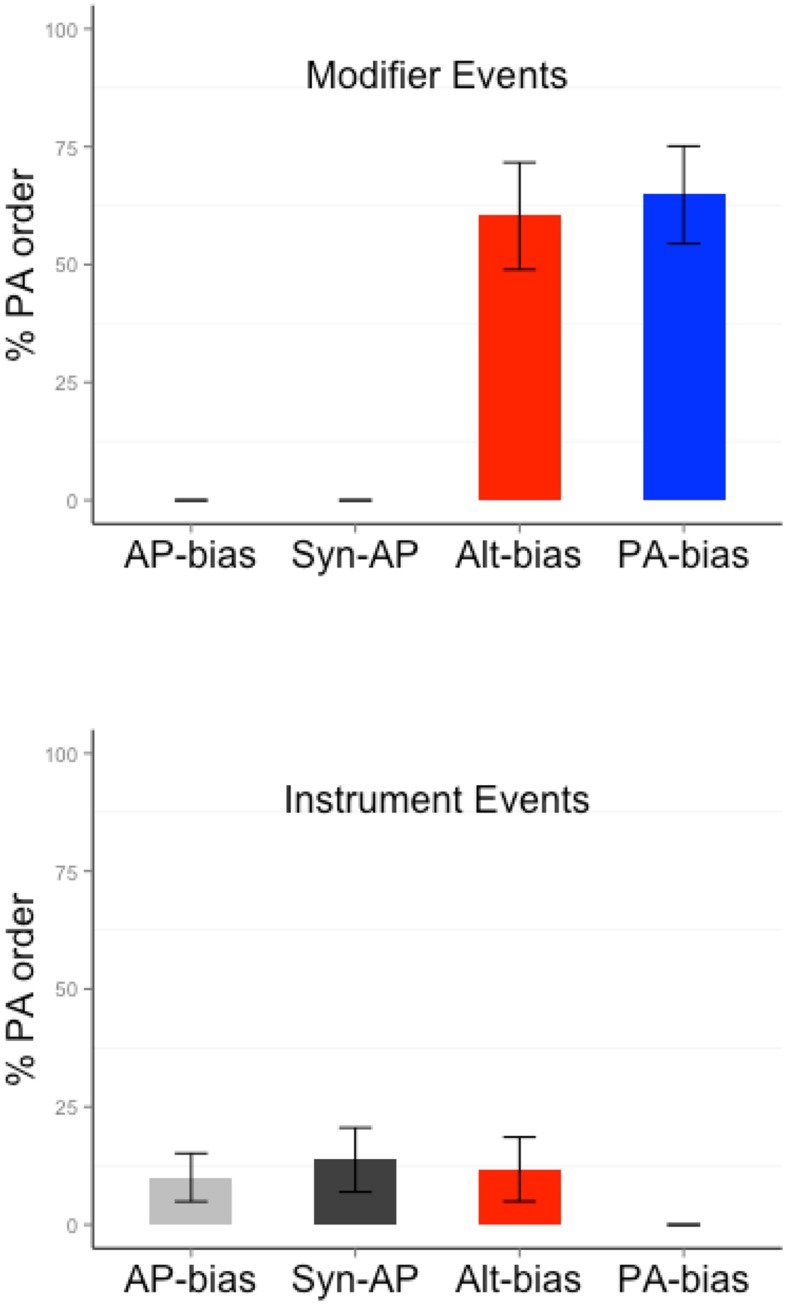
**Proportion of PA order responses produced with different verb and event types in Experiment 2**.

Participants showed an overall preference for AP order [62.38% of the responses contained AP order; 32.11% contained PA order, 5.51% were other, *mu* = 50, *t*(16) = 2.62, *p* = 0.02]. The proportion of PA order for each event type and bias is shown in **Figure 4**. **Table 3** shows the results from the mixed models. The event type analysis for Alt verbs (the only verbs that were tested with both event types) showed a significantly higher likelihood of producing PA order for modifier than instrument events (Wald’s *Z* = 2.37, *p* = 0.02. PA order proportion for Modifier events = 60.29, Instrument events = 11.76). This pattern was consistent with the statistical association between PA order and modifier events and AP order and instrument events during training. The verb bias analysis for modifier events did not reveal any significant differences between PA-only and Alt verbs (Wald’s *Z* = 0.52, *p* = 0.6. PA order proportion for Alt = 60.29, PA-only = 64.78). Similarly, the verb bias analysis for instrument events did not reveal any significant differences between AP-only and Alt/Syn-AP verbs (|*z*| ’s < 0.5, *p*’s > 0.7. PA order proportion for AP-only = 10.03, Syn-AP = 13.76, Alt = 11.76).

**Table 3:** Mixed models of the likelihood of a PA order response in Experiment 2.

	Estimate	*SE*	Wald *Z*	*p*
**Event type analysis**				
**(Alt verbs only)**				
Fixed effects				
Intercept	-9.17	3.34	-2.75	0.006
Modifier (vs. Instrument)	11.31	4.77	2.37	0.02
**Verb bias analyses**				
**Modifier Events**				
Fixed effects				
Intercept	1.18	1.86	0.63	0.53
PA-only (vs. Alt)	0.48	0.92	0.52	0.60
**Instrument events**				
Fixed effects				
Intercept	-8.29	2.46	-3.37	<0.001
Alt (vs. AP-only)	-1.63	4.43	-0.37	0.71
Syn-AP (vs. AP-only)	-0.82	2.58	-0.32	0.75

### Discussion

The main finding from Experiment 2 was that the likelihood of PA order was significantly different when Alt verbs were used to describe modifier events and when the same verbs were used to describe instrument events. This pattern was predicted by the cue validity framework because participants were trained in a language that contained 100% reliable associations between modifier events and PA order, and instrument events and AP order. Thus, this result suggests that the sentence production system can prioritize meaning-to-form mappings when those cues are reliable in the input. The finding cannot be explained by lexicalist frameworks wherein sentences are always constructed via lemma retrieval. If participants relied solely on verb-specific structural preferences, we would have expected similar PA production for the two event types with Alt verbs. On the other hand, constraint-based lexicalist theories, despite their emphasis on rich lexical representations, can accommodate these results because they allow for the co-existence for abstract and lexically specific cues, competition between different cues, and an emergent processing architecture that depends on the reliability of those cues (see e.g., Trueswell and Kim, 1998; Snedeker and Trueswell, 2004). In the case of Alt verbs, the validity of event meaning and individual verbs for predicting sentence form was 100 and 50%, respectively. Thus, mappings between event meaning and structure would be expected to “win” during the process of acquiring the specific language used here. Finally, a dual-path architecture such as the one proposed by Chang et al. (2006) can easily account for the observed pattern because it incorporates both lexically specific and abstract routes to learning and using sentences.

On the flip side of the significant difference between event types for the same verbs, we found no differences between different verbs within each event type that accorded with the lexicalist predictions. However, interpretation of these null effects needs to be qualified because we did not test AP-only and PA-only verbs with the same event types and could therefore not compare them or examine a linear trend as we did in Experiment 1. Thus, although Experiment 2 offers preliminary support for the cue validity framework by demonstrating a significant effect of event type and no effects of verb bias, stronger evidence would come from fully crossing event type and verb bias. We implemented such a design in Experiment 3. Additionally, while Experiment 2 suggests that learners can use event meaning as a cue to sentence structure, it does not clarify whether event meaning was encoded in abstract or verb-specific terms. Because we only tested verbs with their associated event types, participants could have relied on mappings between structure and either abstract thematic roles (e.g., agent, patient) or verb-specific thematic roles (e.g., tickler, ticklee). Experiment 3 provides a stronger test of whether abstract mappings were used because we tested verbs with *unassociated* event types.

## Experiment 3

Experiment 3 employed the same miniature language and procedure as Experiment 2 (**Table 1**). The only difference was that each verb was tested with each event type. This meant that participants were asked to use AP-only verbs to describe instrument events (previously associated) as well as modifier events (previously unassociated) and PA-only verbs to describe modifier events (previously associated) as well as instrument events (previously unassociated). This allowed us to test more comprehensively than in Experiment 2 whether learners relied more on abstract event-to-structure mappings than on verb-specific structural preferences. The strengths of the verb bias manipulations were the same in Experiment 3 as in Experiment 1 (AP-only = 100% AP order, PA-only = 100% PA order, etc.). Therefore, if verb-specific structural preferences influenced production in this language, we would expect gradation in PA order production consistent with verb bias. Alternatively, if the higher predictive validity of abstract meaning-to-form mappings leads the production system to rely on verb-independent and not verb-specific structural information, we would expect no effects of verb bias but an effect of Event Type, with modifier events showing greater likelihood of PA order than instrument events. A third possibility is that both cues influence production even though one cue has higher validity than the other. For example, we might observe higher PA order production for modifier than instrument events as well as (potentially weaker) verb-bias effects.

### Materials and Methods

#### Participants

Twenty right-handed native English speakers from the Washington, D.C. area completed all sessions (18–21 years, mean = 19.15, 17 female). Two participants did not produce verb-first sentence structures and were excluded, leaving a total of eighteen in the final analyses. All participants gave consent under a protocol approved by the Institutional Review Board at The George Washington University. None had participated in the previous experiments.

#### Stimuli

The training stimuli and verb bias manipulations were identical to Experiment 2. Of the 48 test trials, 16 were AP-only, 16 were PA-only, 8 were Alt and 8 were synonymous verbs. Within each verb type, half the trials involved modifier and half involved instrument events. Thus, each combination of verb type and event was tested.

#### Coding

Transcription and coding procedures were identical to the previous experiment. For four randomly selected participants, a different coder transcribed and coded the responses. There was high inter-rater reliability (Cohen’s Kappa = 0.93).

#### Analyses

Experiment 3 employed a fully crossed design (four verb bias conditions tested with each of two event types). Accordingly, the model contained fully crossed effects of Bias and Event Type, plus random effects. The bias and event type factors were dummy-coded with AP-only verbs and Instrument events as the reference level, respectively. A second model was used to examine whether there was a linear AP-only < Alt < PA-only trend in the likelihood of producing PA order (analogous to Experiment 1). This model contained the fixed Bias factor and random effects. The bias factor was coded using orthogonal polynomial coding.

### Results

**FIGURE 5:**
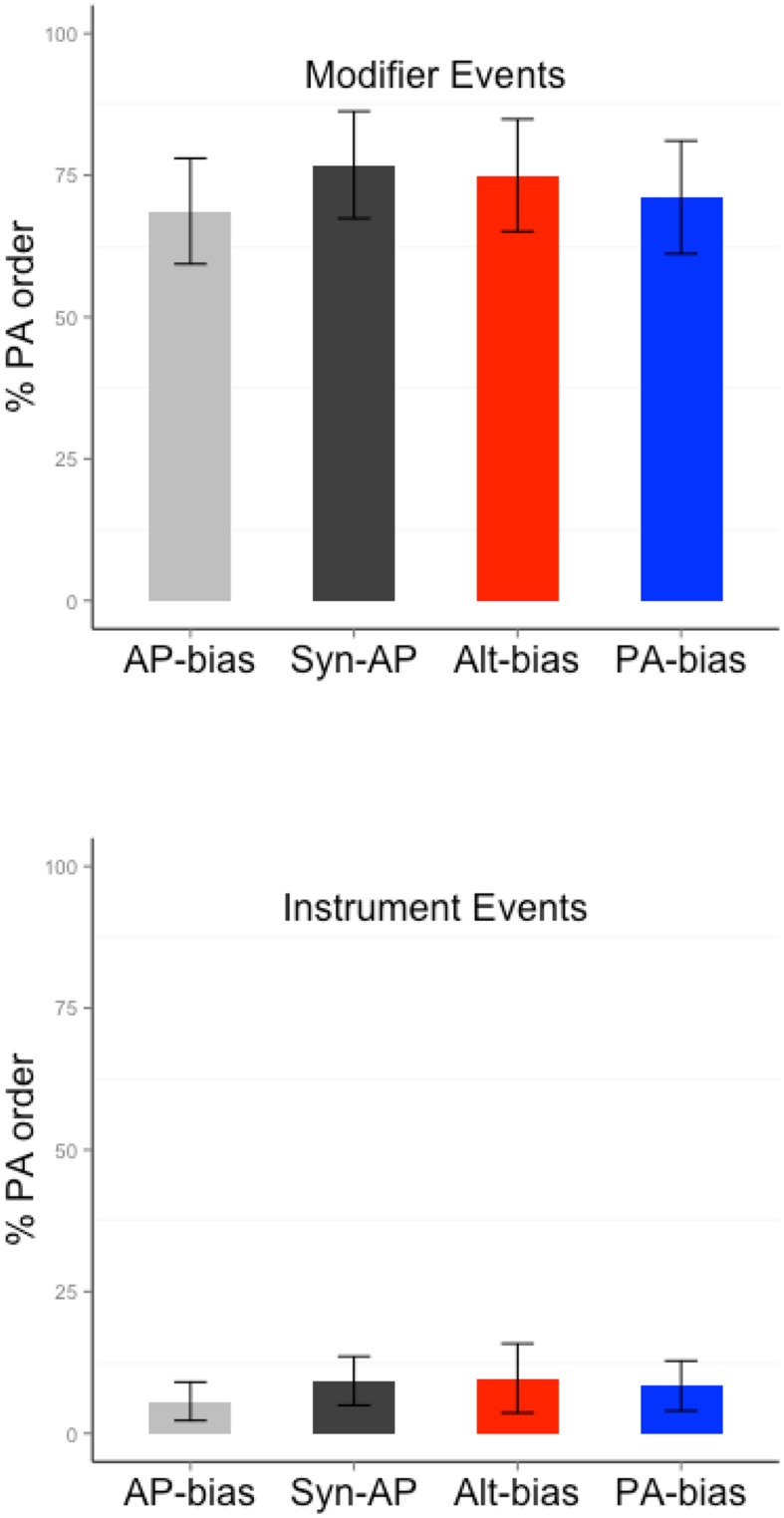
**Proportion of PA order responses produced with different verb and event types in Experiment 3**.

Participants produced more AP than PA responses, but the overall preference for AP order was not statistically significant [56.13% of the responses contained AP order; 36.46% contained PA order, 7.41% were other, *mu* = 50, *t*(17) = 1.4, *p* = 0.18]. The proportion of PA order for each event type and bias is shown in **Figure 5**. In the mixed effects analysis, the full model containing the maximal random effects structure did not converge. Therefore, we simplified the random effects structure in a stepwise fashion (while leaving intact the fixed effects structure). The final model contained random intercepts by participant and item, and random Event Type slope by participant. There was a significantly higher likelihood of producing PA order for modifier than instrument events (Wald’s *Z* = 3.01, *p* = 0.003. PA order proportion for Modifier events = 72.01, Instrument events = 7.67). There were no verb bias effects or interactions (|*z*| ’s < 1.3, *p*’s > 0.2). Relative to AP-only verbs, PA order was no more likely for PA-only, Alt or Syn-AP verbs (PA order proportion for AP-only = 37.03, Syn-AP = 43.45, Alt = 41.96, PA-only = 38.95; **Table 4**).

**Table 4:** Mixed models of the likelihood of a PA order response in Experiment 3.

	Estimate	*SE*	Wald *Z*	*p*
**Fully crossed model**				
Fixed effects				
Intercept	-8.31	2.89	-2.88	0.004
Modifier (vs. Instrument)	11.51	3.82	3.01	0.003
PA-only (vs. AP-only)	0.68	0.66	1.04	0.30
Alt (vs. AP-only)	0.92	0.76	1.21	0.23
Syn-AP (vs. AP-only)	0.80	0.76	1.05	0.30
Interaction (PA-only)	-0.25	0.83	-0.30	0.76
Interaction (Alt)	0.18	1.00	0.18	0.86
Interaction (Syn-AP)	0.64	1.04	0.61	0.54
**AP-only < Alt < PA-only**				
Fixed effects				
Intercept	-1.4	0.73	-1.91	0.06
Linear	0.30	0.44	0.69	0.49
Quadratic	0.12	0.61	0.20	0.84

The model examining gradation based on verb bias did not reveal any significant effects (**Table 4**). Specifically, unlike Experiment 1, there was no linear trend (Wald’s *Z* = 0.69, *p* = 0.49). However, when data from the two experiments were combined, there was no significant interaction of the linear contrast with experiment (Estimate = -0.51, *SE* = 0.40, Wald’s *Z* = -1.27, *p* = 0.21).

### Discussion

Experiment 3 strongly corroborated the patterns found in Experiment 2 by demonstrating a significant effect of event type but not verb bias on word order. This is exactly the pattern predicted by the cue validity approach. Participants in this language were exposed to 100% reliable mappings between event type and structure during training. Subsequently, they employed these mappings to describe modifier events primarily using PA order and instrument events primarily using AP order, across all verb types. The lack of a verb bias effect is intuitively obvious from the patterns of production with AP-only verbs when the event contained a modifier and PA-only verbs when the event contained an instrument (**Figure 5**). In these cases, speakers overrode the verbs’ 100% association with one structure to use it in the opposite structure when such generalization was warranted by the abstract meaning-to-structure mappings. Theories that accord dominance to lexical-structural information would have difficulty in explaining these findings, especially because verb bias was a reliable cue during the learning phase even in this language (e.g., AP-only verbs consistently predicted AP structure, which was in turn consistently and correctly associated with an instrument event). For this reason, the results favor an architecture that clearly separates abstract structural processes from lexical operations (Chang et al., 2006; Konopka and Bock, 2009). Such a separation would better enable the learner to flexibly weight the two routes to sentence production as dictated by the input.

Although the results from Experiment 3 provided no evidence that verb bias influenced production in the given language, they did not unequivocally rule out this possibility. This is because we did not find a significant interaction of the verb bias effect with experiment when we pooled data from Experiments 1 and 3. Thus, it is possible that verb bias exerts a (weak) influence on production even when the language contains an alternative cue with higher validity.

## General Discussion

The three experiments reported here provide evidence that different regularities in the language input can lead to differential use of verb-specific vs. verb-independent information in sentence production. In Experiment 1, verb-specific structural preferences had higher predictive validity than verb-independent mappings during learning. In subsequent production, participants showed sensitivity to the former. In Experiments 2 and 3, verb-independent mappings had higher predictive validity than verb-specific preferences. Under these conditions, participants relied on the abstract mappings. Together, these findings show that learners can acquire new verb-specific structural preferences and verb-general meaning-to-form mappings in a novel language, that they can use either the verb-specific or the verb-general rules to generate new sentences, and that the relative weighting of the two sources during sentence production depends systematically on the statistical properties of the input. The results accord with the predictions of the cue validity framework. They may seem unsurprising *if one assumes a priori* that the language architecture can flexibly adapt to use whichever regularities are reliable in the input. However, as we have reviewed in this paper, this assumption is neither widespread nor well explored empirically. This study was conducted in part to fill this gap.

Broadly, these results add to a rich literature on statistical learning (see Romberg and Saffran, 2010 for a review), which shows that language learners are sensitive to statistical regularities in the input. Such statistical learning extends to the acquisition of new grammatical structures not present in the native language of the learners (Wonnacott et al., 2008, 2012; Perek and Goldberg, 2015). More specifically, we show that the learning system does not attend to and use verb-specific or verb-independent structural information exclusively. Rather, it flexibly learns to rely on whichever regularities have greater predictive validity. Within this perspective, how language is used is inextricably linked to how language is learned. Thus, we believe that our results have implications both for theories of language acquisition and for theories of sentence production.

Debates within language acquisition often pit verb-specific and verb-independent sources of structural information against one another. Different researchers have postulated either that the learning system relies predominantly on verb-specific patterns (Tomasello, 2000, 2003) or that it is guided largely by verb-independent rules (Fisher, 2002a,b). As described before in Introduction, this could be a false dichotomy. Recent evidence suggests that abstract generalizations arise independently of verb-specific frames during development (McClure et al., 2006; Rowland et al., 2012; See also Thothathiri and Snedeker, 2008a). The two routes to sentence production may operate autonomously but together (Chang et al., 2006). One remaining question is how the two routes are weighted at different ages (Rowland et al., 2012). The weighting could be different for children than adults because of differences in the maturity of different brain systems, or alternatively because of differences in the input available at different stages of development [see Rowland et al. (2012) for a more detailed discussion]. The results of the present study suggest that it would be worthwhile to explore the second possibility in more detail. We showed here that adult learners are more or less likely to employ abstract mappings depending on the statistical properties of the input. Future studies could examine the relative validities of different cues in corpora of child-directed vs. adult-directed speech and also compare cue validities for different alternations within a single age group in order to determine whether children’s verb-specific vs. generalization tendencies could be explained systematically using a cue validity framework. Such an approach has been used extensively within the Unified Competition Model to understand sentence comprehension in children and adults [see MacWhinney (2013) for a broad introduction, and Goldberg et al. (2005) and Chan et al. (2009) for empirical demonstrations]. The relevant principles and procedures can be extended to sentence production because language acquisition essentially involves learning to speak via listening to input (Chang et al., 2006).

Within adult language production, it is generally acknowledged that both lexical biases and abstract structural processes could play a role in sentence formulation (e.g., see Konopka and Bock, 2009). However, few studies have examined the circumstances under which these different constraints exert their influence. One clear exception is the study by Wonnacott et al. (2008), which showed that the effect of verb bias on sentence production changes depending on the distribution of verb biases in the input. The researchers contrasted learning in a language in which a majority of the verbs appeared in a single structure (“lexical” language) vs. a language in which a majority of the verbs alternated between two structures (“generalist” language). The results showed that participants were less sensitive to verb bias in the generalist language even when using verbs that individually had as strong a statistical bias for one structure as in the lexical language. This suggested that learners were trading off language-wide distributional cues and item-specific distributional cues in a rational manner (Wonnacott et al., 2008). The current study suggests that there might be a similar trade-off between different *types* of cues, specifically semantic cues involving verb-independent mappings between event meaning and sentence form, and distributional cues linking individual verbs to sentence structures. This in turn raises the question of whether different structural alternations might rely differentially on the two paths to sentence production explored here. While different structures within any alternation usually correspond to some difference in meaning or information structure, there is variability in the *extent* of the meaning difference for different alternations. For example, the causative alternation (“Bob opened the door”/“The door opened”) consists of two structures that are more uncontroversially different in meaning than the dative alternation (“Bob gave Mary the book”/”Bob gave the book to Mary”), which has been argued to consist of structures that are equivalent in meaning under some linguistic frameworks [see Levin (2015) for discussion]. Are speakers more sensitive to verb bias for the latter and more willing to overgeneralize for the former?

The results from the present study provide complementary evidence to that provided by Perek and Goldberg (2015) in two ways. First, we show that speakers’ choices can differentially rely on verb-specific or verb-general structural information based on the properties of the input even when the discourse contexts are not substantively different. Second, all constructions in our miniature languages were verb-initial [cf. verb-final in Perek and Goldberg (2015)]. In the course of incremental sentence processing, earlier arising cues are expected to exert a stronger influence on choice of word order than later arising cues [see Chang (2015) for a computational implementation]. Therefore, our languages are more likely to be biased toward the encoding and using of verb-specific information. Thus, it is particularly notable that speakers in our study overrode verb-specific information and used verb-general mappings instead (Experiments 2 and 3).

This study employed a miniature language paradigm, which allowed us to precisely control the language experience of all the participants and investigate the relationship between input and subsequent production. The sentence structures that participants were trained on in the present study included English nouns and were used in a referential and meaningful context, as in the case of natural language. Thus, while the paradigm is admittedly not entirely naturalistic, the study’s implications are likely to extend to natural language processing.

Across the three experiments, we observed a preference for the AP order (significant in Experiments 1 and 2). This is consistent with an agent-first tendency seen in many of the world’s languages and might reflect a general cognitive bias. However, we cannot rule out the possibility that participants’ English language experience also played a role in this preference.

The interplay between sticking to item-specific experience and generalizing beyond that experience is a persistent topic of research within language. This study showed that learners are more or less willing to generalize when they are exposed to a language containing stronger or weaker evidence for the utility of verb-general meaning-to-form mappings. Future studies can extend these findings by nesting verb-specific patterns within verb-general mappings. This would help clarify whether learners always trade off the two types of cues or whether they can learn a more complex hierarchical pattern that involves both types of information.

## Author Contributions

MT designed the research, analyzed the data, and wrote the paper. MGR collected the data and wrote the paper.

## Conflict of Interest Statement

The authors declare that the research was conducted in the absence of any commercial or financial relationships that could be construed as a potential conflict of interest.

## Footnotes

1 Throughout this article, we assume a unified language system for understanding and producing sentences (Chang et al., 2006). This assumption is supported by behavioral as well as neuroscientific evidence (e.g., see Bock et al., 2007; Silbert et al., 2014). In the current experiments, the testing phase involves only production, but the learning phase, as in real life language acquisition, also involves comprehension. Therefore, we discuss relevant theories that have been proposed within both modalities.

2 The number of excluded participants was much smaller in Experiments 2 and 3 than in Experiment 1. The training and test procedures differed minimally between the experiments. Therefore, we can only speculate as to what other factor could have caused this difference. One possible explanation is that the simple transitive events shown in Experiment 1 were more amenable to being described by the less effortful and English-like AVP order (the predominant pattern seen in the excluded participants).

## Appendix

**Table A1:** Nouns used in training vs. test sessions.

Training	Test
Donkey	Bear
Giraffe	Cat
Lion	Cow
Monkey	Dog
Tiger	Frog
Zebra	Pig
